# Gut Colonization by Methanogenic Archaea Is Associated with Organic Dairy Consumption in Children

**DOI:** 10.3389/fmicb.2017.00355

**Published:** 2017-03-10

**Authors:** Jeroen A. A. van de Pol, Niels van Best, Catherine A. Mbakwa, Carel Thijs, Paul H. Savelkoul, Ilja C. W. Arts, Mathias W. Hornef, Monique Mommers, John Penders

**Affiliations:** ^1^Department of Epidemiology, Care and Public Health Research Institute, Maastricht UniversityMaastricht, Netherlands; ^2^Department of Epidemiology, Grow - School for Oncology and Developmental Biology, Maastricht UniversityMaastricht, Netherlands; ^3^Department of Medical Microbiology, School of Nutrition and Translational Research in Metabolism, Maastricht University Medical CentreMaastricht, Netherlands; ^4^Institute of Medical Microbiology, RWTH Aachen University HospitalAachen, Germany; ^5^Department of Medical Microbiology, Care and Public Health Research Institute, Maastricht University Medical CentreMaastricht, Netherlands; ^6^Maastricht Centre for Systems Biology and Department of Epidemiology, School for Cardiovascular Diseases (CARIM), Maastricht UniversityMaastricht, Netherlands

**Keywords:** microbiota, gut, infant, child, archaea, dairy products, milk, *M. smithii*

## Abstract

The gut microbiota represents a complex and diverse ecosystem with a profound impact on human health, promoting immune maturation, and host metabolism as well as colonization resistance. Important members that have often been disregarded are the methanogenic archaea. Methanogenic archaea reduce hydrogen levels via the production of methane, thereby stimulating food fermentation by saccharolytic bacteria. On the other hand, colonization by archaea has been suggested to promote a number of gastrointestinal and metabolic diseases such as colorectal cancer, inflammatory bowel disease, and obesity. Archaea have been shown to be absent during infancy while omnipresent in school-aged children, suggesting that colonization may result from environmental exposure during childhood. The factors that determine the acquisition of methanogenic archaea, however, have remained undefined. Therefore, we aimed to explore determinants associated with the acquisition of the two main gastrointestinal archaeal species, *Methanobrevibacter smithii* and *Methanosphaera stadtmanae*, in children. Within the context of the KOALA Birth Cohort Study, fecal samples from 472 children aged 6–10 years were analyzed for the abundance of *M. smithii* and *M. stadtmanae* using qPCR. Environmental factors such as diet, lifestyle, hygiene, child rearing, and medication were recorded by repeated questionnaires. The relationship between these determinants and the presence and abundance of archaea was analyzed by logistic and linear regression respectively. Three hundred and sixty-nine out of the 472 children (78.2%) were colonized by *M. smithii*, and 39 out of the 472 children (8.3%) by *M. stadtmanae*. The consumption of organic yogurt (odds ratio: 4.25, CI95: 1.51; 11.95) and the consumption of organic milk (odds ratio: 5.58, CI95: 1.83; 17.01) were positively associated with the presence of *M. smithii*. We subsequently screened raw milk, processed milk, and yogurt samples for methanogens. We identified milk products as possible source for *M. smithii*, but not *M. stadtmanae*. In conclusion, *M. smithii* seems present in milk products and their consumption may determine archaeal gut colonization in children. For the first time, a large variety of determinants have been explored in association with gut colonization by methanogenic archaea. Although more information is needed to confirm and unravel the mechanisms in detail, it provides new insights on microbial colonization processes in early life.

## Introduction

The human gut contains a complex and diverse ecosystem consisting of hundreds of microbial species that are acquired during the first years of life (van Best et al., 2015). Although a myriad of bacterial species have been studied within the human infant gut, important colonizers that are often disregarded are the methanogenic archaea (Horz, 2015). At present, five methanogenic archaea species and two halophilic archaea have been isolated from human feces from which only the *Methanobrevibacter smithii* (*M. smithii), Methanosphaera stadtmanae* (*M. stadtmanae*), and *Methanomassiliicoccus luminyensis* (*M. luminyensis*) have been detected more than once (Miller et al., 1982; Miller and Wolin, 1985; Dridi et al., 2012a; Khelaifia et al., 2013, 2017; Khelaifia and Raoult, 2016). A previous study from Dridi et al. showed that the most dominant archaeal gut inhabitant is *M. smithii* with a prevalence of 88% in children of 0–10 years of age. In contrast, *M. stadtmanae* and *M. luminyensis* tend to colonize the child's gut less frequently with a prevalence of 11 and 1%, respectively (Dridi et al., 2012b).

Upon colonization, methanogenic archaea are responsible for producing the majority of methane in the gut by reducing carbon dioxide into methane in the presence of hydrogen (Roccarina et al., 2010). The hydrogen in the gut is mainly the result of bacterial fermentation, and accumulation of hydrogen subsequently inhibits this process of breaking down food components for energy. Therefore, reduction of hydrogen levels by methanogens stimulates food fermentation by saccarolytic bacteria (Horz and Conrads, 2010; Gaci et al., 2014). On the other hand, colonization by archaea has been suggested to be potentially detrimental for host health due to alterations in gut metabolism and syntrophic interactions with other microbes (Cavicchioli et al., 2003; Conway de Macario and Macario, 2009; Nakamura et al., 2010; Roccarina et al., 2010; Gill and Brinkman, 2011). For instance, in previous studies higher levels of archaea and excreted methane were found in patients with gastrointestinal and metabolic diseases such as colorectal cancer, inflammatory bowel disease, irritable bowel syndrome, constipation, and obesity (Haines et al., 1977; Pimentel et al., 2003; Kim et al., 2012; Blais Lecours et al., 2014; Triantafyllou et al., 2014; Mbakwa et al., 2015; Vandeputte et al., 2016).

Methanogenic archaea have been shown to be absent during infancy while omnipresent in school-aged children and their presence seems to increase with age (Dridi et al., 2012b). The latter suggests that colonization may result either through exposure to sources of archaea during childhood, or through factors shaping the gastrointestinal ecophysiology to make the gut more favorable for archaeal colonization during this time period. The factors that determine the acquisition of methanogenic archaea, however, have remained undefined. Although the rumen of beef cattle have been shown to be a carrier for *M. smithii* and *M. stadtmanae* (Carberry et al., 2014), these human gut colonizers have not been identified in selected food products so far (Brusa et al., 1998). Moreover, no study has conducted a comprehensive analysis on potential lifestyle and dietary determinants of human gut colonization by these methanogenic archaea. Therefore, we aimed to explore a wide variety of potential determinants associated with the acquisition of the two main archaeal species, *M. smithii* and *M. stadtmanae*, in children. To this end, we used extensive data on determinants prospectively gathered through repeated questionnaires within the KOALA Birth Cohort Study in combination with data on presence and abundance of *M. smithii* and *M. stadtmanae* obtained from fecal samples in children of 6–10 years.

## Materials and methods

### Study population

This study was conducted within the KOALA Birth Cohort Study in the Netherlands. The study design has been described in detail elsewhere (Kummeling et al., 2005). In summary, 2,834 pregnant women were recruited between October 2000 and December 2002. Pregnant women with a conventional lifestyle (*N* = 2343) were recruited from an ongoing cohort study on pregnancy-related pelvic girdle pain in the Netherlands. In addition, pregnant women with an “alternative” lifestyle (*N* = 491) were recruited through organic shops, anthroposophical doctors and midwives, anthroposophical under-five clinics, Steiner schools and magazines for special interest groups. The “alternative” lifestyle was expected to differ from the “conventional” lifestyle in vaccination practices, use of antibiotics, dietary habits (breastfeeding, organic foods, and vegetarian diet) and child rearing practices (Kummeling et al., 2005).

**Figure 1:**
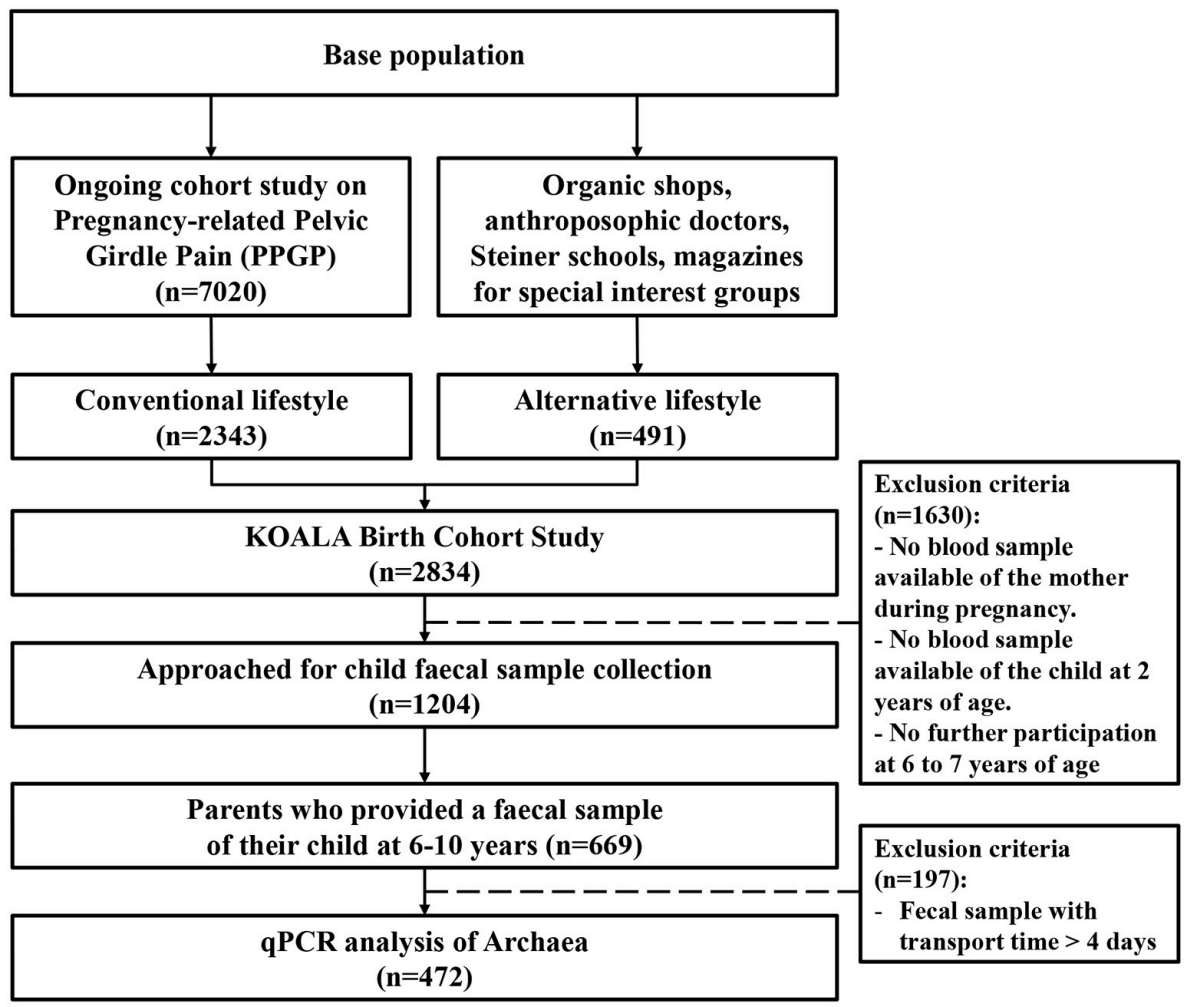
**Flowchart illustrating how the population under study (***n*** = 472) was obtained from the KOALA Birth Cohort Study population**.

Parents of a subgroup of children (*N* = 1,204) were approached for the collection of a fecal sample of their child at age 6–10 (Figure 1). Fecal samples were provided by 669 children. Transport time exceeded 4 days for 197 samples, which were therefore excluded from analyses. As a result, quantitative real-time PCR analysis was performed on 472 fecal samples.

Informed consent was given by all parents, and the study was approved by the Medical Ethics Committee of Maastricht University and the National Ethical Committee for Medical Research.

### Fecal sampling

Parents received a stool sample kit consisting of a feces collection tube with a spoon attached to the lid (Sarstedt, Nürnbrecht, Germany) together with an instruction form on fecal collection. After collection, the fecal sample was sent to the Department of Medical Microbiology at the Maastricht University Medical Center+ by mail. Upon arrival fecal samples were 10-fold diluted in Peptone Water (Oxoid CM0009) containing 20% (vol/vol) glycerol (Merck, Darmstadt, Germany) and stored at −80°C until further analysis.

### Fecal DNA isolation and quantitative real time PCR

Repeated-Bead-Beating (RBB) plus a column-based purification method was used to isolate DNA as described in detail elsewhere (Zoetendal et al., 2006; Salonen et al., 2010). Afterwards, DNA concentration and purity were determined by the Nanodrop 1000 spectrophotometer (Thermo Fisher Scientific, Wilmington, USA). For the enumeration of *M. smithii* and *M. stadtmanae* all fecal samples were subjected to 5′-nuclease based real-time PCR assays. Primers and probes employed for PCR and the amplification and quantification process as performed on an Applied Biosystems Prism 7,000 sequence detection system (Applied Biosystems) have been described in detail elsewhere (Mbakwa et al., 2015). In short, quantification of the abundance of *M. smithii* and *M. stadtmanae* in fecal samples was achieved by comparing the cycle threshold (Ct) values to a standard curve. The standard curves were constructed by subjecting serial 10-fold dilutions of positive plasmid constructs containing the target sequences of *M. smithii* and *M. stadtmanae* to the same qPCR assays. The lower limit of detection was 3.81 log_10_ copies/g feces for *M. smithii* and 4.82 log_10_ copies/g feces for *M. stadtmanae*. Further details on qPCR assays and conditions can be found elsewhere (Mbakwa et al., 2015).

### Determinants

Information on potential determinants was collected by repeated questionnaires in the KOALA Birth Cohort Study. Information was requested from 34 weeks of gestation until 6–7 years of age. Questionnaires covered multiple topics including dietary habits (food frequency questionnaires, FFQ), lifestyle characteristics, hygiene, child rearing practices, diseases, and medication use. Determinants of interest were selected based on findings from previous research on the establishment of the microbiota and the availability of information in repeated questionnaires.

Determinants selected for analysis were: age of the child at time of fecal sample (continuous in years), recruitment group (conventional cohort, alternative cohort), child's gender, maternal education (low: primary school, preparatory vocational or lower general secondary school; middle: vocational, higher general secondary or pre-university; high: higher vocational or academic education), maternal diet during pregnancy (conventional; organic/biodynamic), place, and mode of delivery (vaginal delivery at home; vaginal delivery in hospital; artificially induced delivery in hospital; cesarean section in hospital), gestational age (continuous [weeks]), birth weight (continuous [grams]), regularly cleaning of pacifier in boiling water (no, yes), duration of pacifier use (never, short [until 7 months], long [at least until 12 months]), first time exposure to antibiotics during the first 2 years of life (Never, at 0–3 months, at 3–7 months, at 7–12 months, at 12–24 months), number of siblings at 1 year of age (0, 1, ≥2), regular stay at daycare during the first 2 years of life (never; at host parent or daycare; host parent and daycare combined), vitamin D/AD supplementation during the first 2 years of life (no; yes), number of siblings at 4–5 years of age (0; 1; ≥2), type of pets kept during life of the child (none; dog; cat; other; combination), exposure to farm (animals) during the last 12 months at 6–7 years of age (never; pigs; chicken/pigeons; ox/goat/sheep/horse; children's farm visits; combination), antibiotic use within 1 year prior to fecal sampling (no; yes), child's diet at 6–7 years (conventional; >25% organic/biodynamic), vegetarian diet of child (no; yes), total energy intake (continuous [kcal]), carbohydrate intake (continuous [en%]), protein intake (continuous [en%]), animal protein intake (continuous [en%]), fiber intake (continuous [grams]), regular intake of organic raw vegetables (no; yes), regular intake of organic fruit (no, yes), regular milk consumption (at least once per week; no, yes), regular organic milk consumption (at least once per week; no, yes), regular cheese consumption (at least once per week; no, yes), regular organic cheese consumption (at least once per week; no, yes), regular yogurt consumption (at least once per week; no, yes), regular organic yogurt consumption (at least once per week; no, yes).

### Statistical analysis

Characteristics of the present study population (*N* = 472) and the total KOALA birth cohort (*N* = 2,834) are presented as median plus range for continuous variables and as frequency (n, %) for categorical variables.

Multivariable logistic regression models were used to assess the association between potential determinants and the presence of *M. smithii* and *M. stadmanae*, respectively. Multivariable linear regression models were used to assess the association with *M. smithii* abundance. Variable selection for the regression models was based on purposeful selection as described by Hosmer and Lemeshow (2000). A multivariable model was fitted including all determinants identified from univariable models with a *p* < 0.25. This model was further reduced to only include variables with either a *p*-value lower than 0.10 or variables that affected the parameter estimate (β) of other variables in the model by more than 20%, as recommended by Bursac et al. (2008). This model was subsequently refitted with variables that were not statistically significant (i.e., *p* > 0.25) in univariable analyses. Afterwards, the model was reduced as described before (*p* < 0.10), but only for the added variables. As a result, all variables included in the main effects model were statistically significant or made an important contribution based on the presence of other variables in the model (Bursac et al., 2008). After completion of purposeful selection the resulting main effects model was additionally adjusted for the following covariables of prior interest: age at time of fecal sampling (years), gender (male, female), total energy intake (kcal), recruitment group (conventional, alternative), and BMI (z-score). For determinants with more than 30 missing values a missing value group was constructed. Models were checked for multicollinearity through Variance Inflation Factor (VIF) scores. If VIF scores exceeded 10, determinants causing multicollinearity were separated into independent models to obtain effect estimates without multicollinearity for the affected determinants. To limit the number of spurious associations, results from multivariable analyses were corrected for multiple comparisons by adjusting α by Benjamini-Hochberg procedure at a false discovery rate (FDR) level of *q* = 0.05 (Benjamini and Hochberg, 1995).

In secondary analyses, the impact of the number of consumed organic dairy products, and organic as well as raw vegetable and fruit products on the presence and abundance of *M. smithii* and *M. stadtmanae* was examined. For this purpose the individual variables for organic dairy (cheese, milk, and yogurt) and organic fruit and raw vegetables were grouped into index variables as follows: number of different organic dairy products (0; 1; 2; 3), number of different organic raw vegetable and fruit products (0; 1; 2). Since organic dairy products were significantly associated with the presence of *M. smithii*, analyses were extended to yogurt, milk and cheese, irrespective of organic or conventional origin.

For all statistical analyses IBM SPSS version 23 (SPSS Inc., Chicago, IL) was used.

### Verification of archaeal presence in milk

#### Raw bovine milk in tanker trucks

To validate findings from this study, publicly available data from another study was used to identify archaea in 975 raw milk samples collected from 899 tanker trucks (Kable et al., 2016). These individual trucks arrived at two dairy processors in San Joaquin Valley of California for product manufacturing during three seasons between October 2013 and September 2014. The sequences of the 16S rRNA V4 regions were obtained via the Qiita database (https://qiita.ucsd.edu) under study ID 10485, and further analyzed within Qiime (Caporaso et al., 2010).

#### Metagenomic DNA isolation from dairy products

Processed milk and yogurt samples were collected from biodynamic, organic and non-organic brands from the supermarket (Supplementary Table 1). Raw unprocessed milk was obtained from a local farmer via a standard tap-procedure (Brunimat). Metagenomic DNA was extracted from dairy products with the PowerFood™ Microbial DNA Isolation kit (MoBio Laboratories Inc.) according to manufacturer's instructions as evaluated in detail elsewhere (Quigley et al., 2012). In short, cell lysis with chaotrophic agents and bead-beating plus a column-based extraction was used to isolate DNA.

## Results

Fecal samples from 472 children with a median age of 7.2 (6.0–12.0) years were analyzed. Of these, 369 (78.2%) out of 472 were colonized by *M. smithii*, while 39 (8.3%) out of 472 children were colonized by *M. stadtmanae*. Due to the low colonization rate of *M. stadtmanae*, analyses on its abundance were not performed in this study. Supplementary Table 2 shows participant characteristics of the KOALA Birth Cohort Study (*n* = 2834) and the study population (*n* = 472) for all selected determinants. In general, the distribution of participant characteristics of the study population was similar to the population of the entire KOALA Birth Cohort Study.

### *Methanobrevibacter smithii* presence and abundance

The multivariable regression models (Table 1 and Supplementary Table 3) showed a positive association between organic yogurt and organic milk consumption and *M. smithii* colonization. Children who regularly consumed organic yogurt were more than four times as likely to be colonized by *M. smithii* as compared to children who did not consume organic yogurt [OR_adjusted_: 4.25 95% Confidence Interval (CI95): 1.51; 11.95], whereas children consuming organic milk were even over five times more likely to be colonized by *M. smithii* as compared to children who did not consume organic milk (OR_adjusted:_ 5.58, CI95: 1.83; 17.01). We subsequently performed secondary analyses to examine the effect of the number of different organic dairy products being consumed on *M. smithii* colonization (Table 2 and Supplementary Table 4). These analyses showed a statistically significant increasing trend between the number of dairy products consumed and the chance of colonization by *M. smithii* (Table 2, *p* = 0.002).

**Table 1:** **Final multivariable logistic regression model showing the association between potential determinants and the presence of ***Methanobrevibacter smithii*****.

***M. smithii*** **presence**			
**Adjusted main effects model (*****N*** = **419)**^a^			
**Determinant**	**OR (CI95%)**	**FDR crit.^b^**	***p*-value**
Regular intake of organic milk^c^^,^^d^			
No	Ref.		
Yes	5.58 (1.83; 17.01)	0.006	**0.003**^f^
Regular intake of organic yogurt^c^^,^^d^			
No	Ref.		
Yes	4.25 (1.51; 11.95)	0.006	0.006
Diet of child^e^			
Conventional (≤ 25% organic)	Ref.		
Organic (incl. biodynamic; >25% organic)	0.36 (0.17; 0.79)	0.011	**0.010**^f^
Fiber intake (g)^e^	0.95 (0.88; 1.03)	0.028	0.250

a *Model adjusted for: age at fecal sampling (years), gender (male/female), recruitment group (conventional/alternative), total energy intake (kcal), and BMI (z-score)*.

b *Critical FDR cut-off level as determined by Benjamini-Hochberg procedure*.

c *Missing value category omitted (included in FDR correction)*.

d *Due to multicollinearity both regular intake of organic milk and regular intake of organic yogurt were included in separate models, which included all other variables as listed in this table*.

e *Parameter estimates presented for the model containing organic milk intake. Parameter estimates did not change substantially when the variable “organic milk intake” was replaced by the variable “organic yogurt intake” (data not shown)*.

f *Significant association after correction for FDR by Benjamini-Hochberg procedure, also indicated in bold*.

**Table 2:** **Secondary multivariable logistic regression model estimating the impact of the number of organic dairy products on the presence of ***Methanobrevibacter smithii*****.

***M. smithii*** **presence**			
**Adjusted main effects model (*****N*** = **406)**^a^			
**Determinant**	**OR (CI95%)**	**FDR crit.^b^**	***p*-value**
First exposure to antibiotics (during first 2 years of life)			
Never	Ref.		
At 0–7 months	0.61 (0.33; 1.13)	0.020	0.115
At 8–12 months	1.20 (0.58; 2.49)	0.035	0.631
At 13–24 months	0.78 (0.39; 1.57)	0.030	0.490
Regular intake of organic products (cheese, milk, and yogurt)			
Trend (0, 1, 2, 3)	2.12 (1.31; 3.43)	0.005	**0.002**^c^
Diet of child			
Conventional (≤ 25% organic)	Ref.		
Organic (incl. biodynamic; >25% organic)	0.30 (0.14; 0.65)	0.010	**0.002**^c^

a *Model adjusted for: age at fecal sampling (years), gender (male/female), recruitment group (conventional/alternative), total energy intake (kcal) and BMI (z-score)*.

b *Critical FDR cut-off level as determined by Benjamini-Hochberg procedure*.

c *Significant association after correction for FDR by Benjamini-Hochberg procedure, also indicated in bold*.

For all other potential determinants that have been examined in the present study, no relationship with *M. smithii* colonization was found. Moreover, when examining the relationship between *M. smithii* colonization and the consumption of dairy products, irrespective of organic or conventional origin, we did neither find an association for milk nor for yogurt and nor for cheese consumption.

In contrast to the analyses on the presence of *M. smithii*, none of the determinants assessed in this study were positively associated with the abundance of *M. smithii* (Supplementary Tables 5, 6).

In models for both *M. smithii* presence and abundance the model adjustment by a priori selected covariates did not meaningfully alter parameter estimates. The minimal effect of these covariates on parameter estimates indicates that models for *M. smithii* were robust.

### *Methanosphaera stadtmanae* presence

Multivariable regression analyses on the presence of *M. stadtmanae* showed no significant results after FDR correction (Supplementary Table 7). However, after adjustment for a priori selected covariables birth by cesarean section (OR: 6.89, CI95: 2.09; 22.67), first exposure to antibiotics at 13–24 months (OR: 3.38, CI95: 1.34; 8.50) and organic fruit intake (OR: 4.73, CI95: 1.64; 13.62) were associated with an increased *M. stadtmanae* presence (Table 3). In secondary analyses to examine the effect of the number of organic fruit and vegetable products consumed on the presence of *M. stadtmanae* no significant trend was found (*p* = 0.321).

**Table 3:** **Final multivariable logistic regression model showing the association between potential determinants and the presence of ***Methanosphaera stadtmanae*****.

***M. stadtmanae*** **presence**			
**Adjusted main effects model (*****N*** = **420)**^a^			
**Determinant**	**OR (CI95%)**	**FDR crit.^b^**	***p*-value**
Birthweight (g)^c^	1.001 (1.000; 1.002)	0.019	0.028
Place and mode of delivery^c^			
Natural birth at home	Ref.		
Natural/artificial birth at hospital	1.61 (0.67; 3.88)	0.034	0.288
Caesarean section	6.89 (2.09; 22.67)	0.003	**0.002**^f^
First exposure to antibiotics (during first 2 years of life)^c^			
Never	Ref.		
At 0–7 months	0.54 (0.16; 1.83)	0.038	0.323
At 8–12 months	0.70 (0.21; 2.36)	0.050	0.566
At 13–24 months	3.38 (1.34; 8.50)	0.013	**0.010**^f^
Regular intake of organic milk^d^^,^^e^			
No	Ref.		
Yes	3.52 (0.97; 12.73)	0.022	0.056
Regular intake of organic yogurt^d^^,^^e^			
No	Ref.		
Yes	1.690 (0.49; 5.86)	–	0.409
Regular intake of organic fruit^c^			
No	Ref.		
Yes	4.73 (1.64; 13.62)	0.006	**0.004**^f^
Animal protein intake (en%)^c^	0.77 (0.65; 0.92)	0.009	**0.004**^f^

a *Model adjusted for: age at fecal sampling (years), gender (male/female), recruitment group (conventional/alternative), total energy intake (kcal) and BMI (z-score)*.

b *Critical FDR cut-off level as determined by Benjamini-Hochberg procedure*.

c *Parameter estimates presented for the model containing organic milk intake. Parameter estimates did not change substantially when the variable “organic milk intake” was replaced by the variable “organic yogurt intake” (data not shown)*.

d *Missing value category omitted (included in FDR correction)*.

e *Due to multicollinearity both regular intake of organic milk and regular intake of organic yogurt were included in separate models, which included all other variables as listed in this table*.

f *Significant association after correction for FDR by Benjamini-Hochberg procedure, also indicated in bold*.

In models for *M. stadtmanae* presence minor differences in model estimates were found after adjustment for a priori selected covariates. These differences may indicate underlying relationships between potential determinants and a priori selected covariates or model instability of *M. stadtmanae* models.

### Verification of archaeal presence in milk

**Figure 2:**
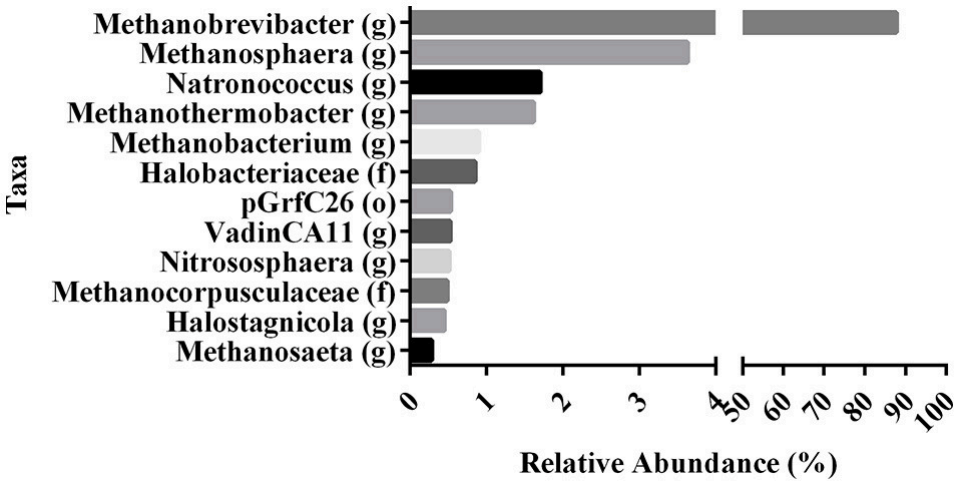
**Variation in the relative abundances of dominant archaea taxa in raw milk of re-analyzed 16S sequence-data from Kable et al. (2016)**. Data represent the abundances of the various archaeal taxa (found at 0.1% or greater) as proportion of the total archaeal populations in raw tanker milk. Relative abundances of OTU's rarified at 15,000 sequences per sample are indicated at their highest identified taxa. (o), order. (f), family. (g), genus.

**Figure 3:**
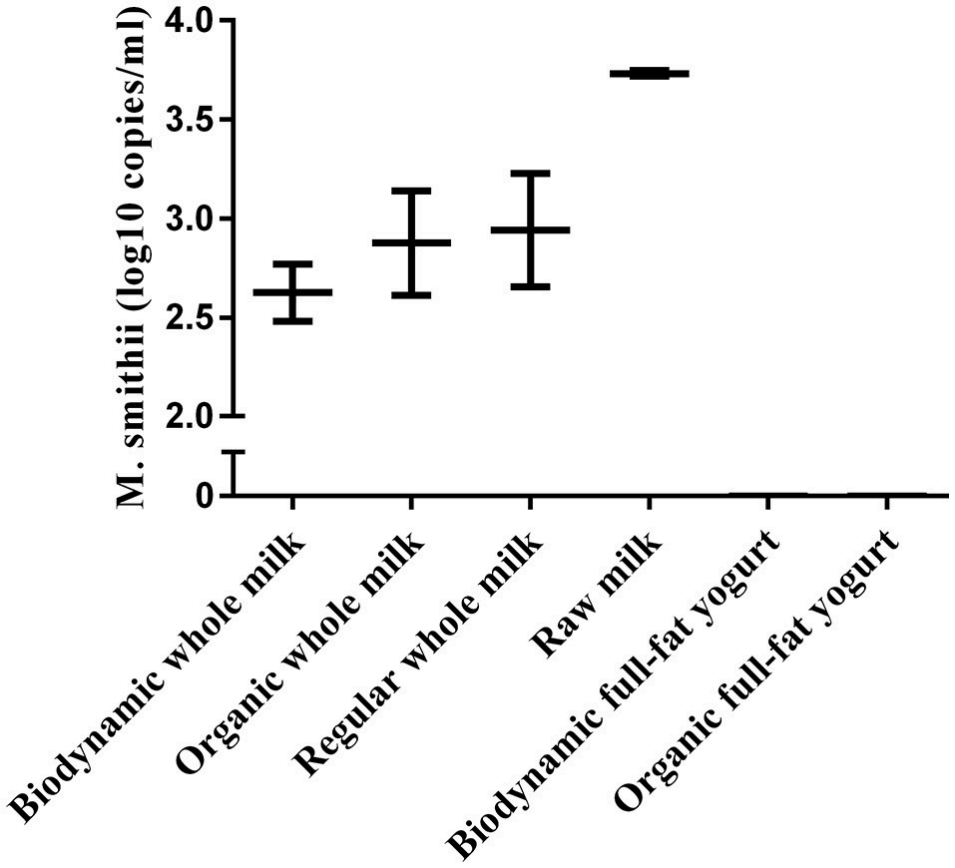
**Absolute counts of ***M.smitthii*** in whole or full-fat dairy products (***n*** = 2–3)**. Replicates (*n*) denotes repeated isolation and quantification of the product. Average counts were calculated from archaea positive samples only.

To evaluate the contribution of milk and yogurt to gut colonization by archaea, we re-analyzed 16S sequence-data of raw milk from a previous study (Kable et al., 2016) as only the bacterial results of these samples from 899 tanker trucks were reported in the original publication. The relative abundances showed that the vast majority of all recovered archaeal sequences could be assigned to the genera *Methanobrevibacter* (87.70%) and *Methanosphaera* (3.63%) compared to the other 10 (<1.70%; Figure 2). In addition, 947 out of the 975 milk samples (97.13%) were positive for Methanobrevibacter while Methanosphaera sequences were present in 348 of the milk samples (35.69%). To further assess the absolute counts of methanogens in dairy products, we subsequently screened unprocessed raw milk, pasteurized milk, and pasteurized yogurt samples (Supplementary Table 1). *M. smithii* and *M. stadtmanae* were quantified by qPCR on isolated DNA of these samples. Although *M. smithii* could not be detected in yogurt samples, we found significant levels of *M. smithii* in milk (Figure 3). The average absolute counts in processed milk was highly similar for biodynamic (2.63 log10 DNA copies/ml), organic (2.88 log10 DNA copies/ml) and conventional milk (2.94 log10 DNA copies/ml). However, raw milk (3.73 log10 DNA copies/ml) showed higher *M. smithii* counts compared to processed milk. For *M. stadtmanae*, no detectable levels could be measured in any of the samples. In conclusion, we identified milk as a possible source off *M. smithii*.

## Discussion

To the best of our knowledge, this is the first large-scale explorative study investigating the associations between a wide range of potential determinants and intestinal colonization by methanogenic archaea in school-aged children. We found that consumption of organic yogurt and milk products is related to colonization of *M. smithii* in particular. Moreover, we identified *M. smithii* in raw and processed milk, which could therefore be considered as potential sources for archaeal colonization.

The difference in metabolic activity might be a possible explanation for the large difference in the prevalence of the two main intestinal archaea. *M. smithii* is responsible for producing the majority of methane in the gut by reducing carbon dioxide using hydrogen as the primary electron donor. Alternatively, *M. smithii* can use formate as a direct substrate to produce methane (Roccarina et al., 2010). In contrast, *M. stadtmanae* is only able to produce methane from hydrogen (Gaci et al., 2014). Moreover, colonization by *M. smithii* and *M. stadtmanae* might originate from different environmental sources, which could explain their different prevalence. Factors that play a role in the acquisition of methanogenic archaea are largely unknown. However, the environment is assumed to play a crucial role in gut colonization. This view is strengthened by a twin study that indicated that not genetic factors, but shared and unique environmental factors were of importance for the occurrence of methanogens in humans (Florin et al., 2000). The prospective design and long-term follow-up allowed us to examine a wide range of environmental, dietary and life-style associated determinants throughout childhood. Out of the determinants present during infancy, caesarean section compared to vaginal delivery at home, first exposure to antibiotics in the 2nd year of life compared to no antibiotic treatment during the first 2 years and organic fruit consumption were associated with increased odds of colonization by *M. stadtmanae*. These findings on *M. stadtmanae* in the present study need however to be interpreted with great care. Due to the small group of children colonized by *M. stadtmanae* (*n* = 39), models for *M. stadtmanae* were prone to instability. Therefore, these factors need to be verified in other studies with, preferably, larger sample sizes. In particular, the association with birth mode is in contrast with previous findings that intestinal colonization by methanogenic archaea in neonates and infants is very rare (Palmer et al., 2007). However, it could be postulated that the pioneer species associated with caesarian section delivery drive a subsequent colonization pattern that is more favorable for the establishment of *M. stadtmanae* at a later stage. As such, it is important to study the co-occurrence and co-exclusion of archaea and bacterial taxa in future studies, in order to identify microbial networks that might favor archaeal colonization.

Although one previous study found females to be more often colonized by methanogenic archaea than males (Florin et al., 2000), the current view is that there is no association between methanogenic archaea and gender (Dridi et al., 2011b). In this study, we assessed fecal samples of 244 (51.7%) males and 228 (48.3%) females. Of all males under study 187 (76.6%) were colonized by *M. smithii*, while 182 (79.8%) females were colonized by *M. smithii*. Furthermore, 19 (7.8%) males were colonized by *M. stadtmanae*. This was similar to the colonization by *M. stadtmanae* in females, as 20 (9.6%) females were colonized. Moreover, we also did not find an association between gender and either *M. smithii* or *M. stadtmanae*.

So far, only one study assessed potential carriers of archaea in food, but yogurt and milk have not been assessed herein (Brusa et al., 1998). The present study suggests an association between dietary factors and archaeal colonization, as organic yogurt and milk were significantly associated with the presence of *M. smithii*. Due to the high correlation between organic milk and yogurt intake, we were however not able to disentangle whether only organic milk or organic yogurt or both were truly responsible for the association with *M. smithii* during multivariable analyses. Cheese was however not associated with *M. smithii* colonization, which is in line with previous findings where no *M. smithii* or *M. stadtmanae* were detected in different types of cheese (Brusa et al., 1998). Analyses on the number of dairy products consumed, used to address multicollinearity, indicated that the consumption of multiple different dairy products might be associated with an increased *M. smithii* presence.

A well-known carrier of *M. smithii* and *M. stadtmanae* is the rumen of beef and cows (Carberry et al., 2014; Cersosimo et al., 2016). Therefore, it is likely that products derived from cows, such as dairy products, may contain some of these taxa, which was reflected in our results. Moreover, these specific methanogenic archaea have also been found in soil which could be the route of origin to cows as revealed from the Integrated Microbial Next Generation Sequencing database (Lagkouvardos et al., 2016). The archaeal presence in soil might be the underlying reason that especially organic products were associated with colonization. Organic cows have more outdoor access compared to conventionally farmed cows and could therefore have increased archaea uptake. Additionally, drugs for organic cows are less prescribed whereas conventional cows more often receive antibiotics to prevent microbial infections (Zwald et al., 2004). Methanogens are susceptible to antibiotics such as bacitracin, a commonly used antibiotic in cattle, which might eliminate them (Dridi et al., 2011a). Although the screening of multiple dairy products indicated the presence of *M. smithii* in both conventional and organic dairy products, the latter could alternatively explain the lack of association of consumption of conventional dairy products with *M. smithii* colonization within our cohort study.

As the culture-independent techniques applied in the present study do not distinguish viable microbes from cell-free DNA originating from lysed microorganisms, we cannot completely exclude that dairy products only contain archaeal DNA instead of viable archaea. However, we used the concentrated pellet of the microbial cells for downstream analyses of milk products, thereby minimizing the detection of circulating cell-free DNA. It is therefore likely that a living fraction of archaeal cells has been measured.

For milk, the typical neutral pH is viable for both *M. smithii* and *M. stadtmanae*, which favor an optimal pH of 6.9–7.4 whereas yogurt has a more acidic environment (Miller and Lin, 2002; Ledenbach and Marshall, 2009; Dridi et al., 2011b), which might explain why we could not detect methanogenic archaea in yogurt samples. In addition, methanogens have been discovered in a wide variety of extreme environments with temperatures until at least 100°C (Elias et al., 1999; Tung et al., 2005). The latter might indicate that these microbes could resist heat treatments and pasteurization performed for milk products. All in all, this strengthens the possibility that viable methanogenic archaea could indeed be present in milk products, even after thermal processing.

To get definite prove that milk products are a source of archaea, future studies using either culture-based methods or molecular methods that enable differentiation between intracellular and cell-free DNA, such as the use of propidium monoazide (PMA) as a membrane impermeable DNA intercalating dye (Janssen et al., 2016), are warranted. Several studies already identified living bacteria with an implemented PMA-assay in both human feces and processed milk (Bae and Wuertz, 2009; Soejima et al., 2012; Quigley et al., 2013; Cangelosi and Meschke, 2014).

In conclusion, dairy products, in particular organic milk products, may play an influential role in the colonization of the gut by *M. smithii* in children. Moreover, *M. smithii* seems to be present in both raw and commonly consumed milk products. For the first time, a large variety of determinants have been explored in association with gut colonization by methanogenic archaea. Although more information is needed to confirm and unravel the mechanisms of archaeal colonization in more detail, it may provide new targets for prevention of diseases associated with the presence of methanogenic archaea.

## Author contributions

Jv performed the analyses on the cohort, and wrote partly the manuscript together with Nv. Nv designed the laboratory experiments, performed laboratory isolation, and qPCR techniques on milk products, analyzed the genomic data, and wrote partly the final manuscript as submitted. CM performed the laboratory isolation of fecal DNA and qPCR techniques, data processing, and approved the final manuscript as submitted. JP contributed to the design of the study and collection of the data, supervised the laboratory work, interpreted the data, critically reviewed the manuscript, and approved the final manuscript as submitted. MM contributed to the design of the study and collection of the data, interpreted the data, critically reviewed the manuscript and approved the final manuscript as submitted. CT and IA contributed to the design of the study and collection of the data, critically reviewed and revised the manuscript and approved the final manuscript as submitted. PS and MH critically reviewed and revised the manuscript and approved the final manuscript as submitted.

## Funding

This work was supported by TI Food and Nutrition, public private partnership in food and nutrition research. The mission is to contribute to optimum human nutrition, food safety, and sustainable food production and to increase the competitiveness of the food industry. Partners are key players in the global food industry, leading research institutes, universities, and medical centers. Additional funding for data collection was received from: Netherlands Organization for Health Research and Development (grant 2100.0090), Netherlands Asthma Foundation (grants 3.2.03.48, 3.2.07.022), Netherlands Heart Foundation (grant 2008B112), Triodos Foundation, Phoenix Foundation, Raphaël Foundation, Iona Foundation, Foundation for the Advancement of Heilpedagogiek, Royal Friesland Foods (currently FrieslandCampina), Netherlands Sugar Foundation, and the Ministry of Economic Affairs. The sponsors had no influence on the analysis and reporting of this study. This work has also been supported by the Deutsche Forschungsgemeinschaft (Ho2236/8-1 and Priority Program 1656 to MH) and by ZonMW/JPI HDHL Intestinal Microbiomics (grant 50-52905-98-599 to JP).

### Conflict of interest statement

The authors declare that the research was conducted in the absence of any commercial or financial relationships that could be construed as a potential conflict of interest.
