# Supplementary material for: Gut Colonization by Methanogenic Archaea Is Associated with Organic Dairy Consumption in Children
Source: Front Microbiol. 2017 Mar 10;8:355. doi: 10.3389/fmicb.2017.00355 (PMC5344914; doi:10.3389/fmicb.2017.00355)
Supplement: Supplementary file 1 [file DataSheet1.DOCX]

Supplementary Material

Gut colonization by methanogenic archaea is associated with organic dairy consumption in children

Jeroen A.A. van de Pol^1,2,†^, Niels van Best^3,4,†^, Catherine Akwi Mbakwa^1^, Carel Thijs^1^, Paul H. Savelkoul^3,5^, Ilja C.W. Arts^6^, Mathias W. Hornef^4^, Monique Mommers^1‡^, John Penders^3,5‡*^

^1^Care and Public Health Research Institute (Caphri), Department of Epidemiology, Maastricht University, Maastricht, the Netherlands

^2^School for Oncology and Developmental Biology (GROW), Department of Epidemiology, Maastricht University, Maastricht, the Netherlands

^3^School of Nutrition and Translational Research in Metabolism (NUTRIM), Department of Medical Microbiology, Maastricht University Medical Centre+, Maastricht, the Netherlands

^4^Institute of Medical Microbiology, RWTH Hospital RWTH Aachen, Aachen, Germany

^5^Care and Public Health Research Institute (Caphri), Department of Medical Microbiology, Maastricht University Medical Centre+, Maastricht, the Netherlands

^6^School for Cardiovascular Diseases (CARIM), Maastricht Centre for Systems Biology (MaCSBio) and Department of Epidemiology, Maastricht University, Maastricht, the Netherlands

^†^ These first authors contributed equally to this work
^‡^ These last authors contributed equally to this work

*** Correspondence:**Corresponding Author
j.penders@maastrichtuniversity.nl

# Supplementary Figures and Tables

**Supplementary Table 1.** Absolute counts of *M. smithii* and *M. stadmanae* in dairy whole/full-fat products (n=2-3). *log10 DNA copies/ml, average. Replicates (*n)* denotes repeated isolation and quantification of the product. Average counts were calculated from positive samples only.

| **Dairy Product** | **M.smithii counts*** | **M.stadmanae counts*** | **Thermal Processing** | **Expiration (days)** | **Origin** |
| --- | --- | --- | --- | --- | --- |
| Biodynamic milk | 2.63 | - | Pasteurised | 4 | Zuiver Zuivel |
| Organic milk | 2.88 | - | Pasteurised | 5 | Weerribben Zuivel |
| Regular milk | 2.94 | - | Pasteurised | 1-5 | Campina & Plus |
| Raw milk | 3.73 | - | None | - | Local Farmer |
| Organic yogurt | - | - | Pasteurised | 14 | Weerribben Zuivel |
| Biodynamic yogurt | - | - | Pasteurised | 15 | Zuiver Zuivel |

**Supplementary Table 2.** Patient characteristics of the KOALA birth cohort study and the population under study

| **Determinants** | **KOALA Birth Cohort Study (n = 2834)** | **Study Population**  **(n = 472)** |
| --- | --- | --- |
| **Continuous variables** | **Median (range)** | **Median (range)** |
| General characteristics |  |  |
| *Age at fecal sampling (years) ^b^* | - | 7.2 (6.0 – 10.0) |
| *Birthweight (g)* | 3500 (780 – 5455) | 3570 (2140 – 5000) |
| *Gestational age (weeks)* | 40 (26 – 43) | 40 (35 – 42) |
| *Breastfeeding (months)* | 4 (0 – 13) | 5 (0 – 13) |
| *BMI (z-score) ^b^* | - | -0.2 (-2.7 – 2.8) |
| Dietary intake (per day) |  |  |
| *Total energy intake (kcal)* | 1437.5 (740.0 – 2844.1) | 1419.5 (788.3 – 2415.2) |
| *Carbohydrate intake (en%)* | 55.7 (37.1 – 74.5) | 55.7 (42.3 – 69.5) |
| *Protein intake (en%)* | 14.6 (8.0 – 21.9) | 14.5 (8.8 – 20.4) |
| *Animal protein intake (en%)* | 9.0 (1.6 – 16.3) | 9.0 (2.2 – 15.3) |
| *Fiber intake (g)* | 15.1 (5.4 – 31.4) | 15.4 (6.1 – 28.1) |
| **Categorical variables** | **N (%)^a^** | **N (%)^a^** |
| Gender |  |  |
| *Male* | 1451 (51.3) | 244 (51.7) |
| *Female* | 1376 (48.7) | 228 (48.3) |
| Recruitment group |  |  |
| *Conventional* | 2343 (82.7) | 354 (75.2) |
| *Alternative* | 491 (17.3) | 117 (24.8) |
| Maternal education *^c^* |  |  |
| *Lower education* | 289 (10.7) | 34 (7.5) |
| *Medium education* | 1060 (39.4) | 171 (37.7) |
| *Higher education* | 1341 (49.9) | 248 (54.7) |
| Older siblings (at 1 year of age) |  |  |
| *None* | 1193 (44.1) | 187 (39.7) |
| *One* | 1110 (41.0) | 206 (43.7) |
| *Two or more* | 404 (14.9) | 78 (16.6) |
| Younger and older siblings (at 4-5 years of age) |  |  |
| *None* | 199 (9.7) | 46 (10.3) |
| *One* | 1162 (56.8) | 245 (54.9) |
| *Two or more* | 685 (33.5) | 155 (34.8) |
| Regular visits to daycare or stay at a host parent (during the first 2 years of life) |  |  |
| *No* | 960 (35.2) | 161 (34.3) |
| *Host parent* | 680 (24.9) | 122 (26.0) |
| *Daycare or daycare and host parent* | 1086 (39.8) | 187 (39.8) |
| Place and mode of delivery |  |  |
| *Natural birth at home* | 1187 (44.9) | 219 (47.1) |
| *Natural/artificial birth at hospital* | 1147 (43.4) | 198 (42.6) |
| *Caesarean section* | 311 (11.8) | 48 (10.3) |
| First exposure to antibiotics (during first 2 years of life) |  |  |
| *Never* | 1074 (43.5) | 212 (46.7) |
| *At 0-7 months* | 562 (22.8) | 92 (20.3) |
| *At 8-12 months* | 404 (16.4) | 75 (16.5) |
| *At 13-24 months* | 430 (17.4) | 75 (16.5) |
| Use of antibiotic treatments (1 year before fecal sampling) *^b^* |  |  |
| *No* | - | 396 (85.2) |
| *Yes* | - | 69 (14.8) |
| Duration of pacifier use by the child *^d^* |  |  |
| *Never* | 684 (27.5) | 127 (27.6) |
| *Early* | 587 (23.6) | 108 (23.5) |
| *Late* | 1214 (48.9) | 225 (48.9) |
| Cleaning of pacifier in boiling water |  |  |
| *Never/Sometimes* | 458 (22.0) | 84 (22.3) |
| *Always* | 1624 (78.0) | 292 (77.7) |
| Presence of pets at 6 to 7 years of life. |  |  |
| *None* | 928 (47.0) | 217 (46.7) |
| *Dog* | 227 (11.5) | 44 (9.5) |
| *Cat* | 242 (12.3) | 58 (12.5) |
| *Other* | 338 (17.1) | 90 (19.4) |
| *Combination* | 236 (12.0) | 56 (12.0) |
| Monthly exposure to farm (animals) at 6-7 years of age |  |  |
| *No* | 1309 (72.7) | 303 (72.8) |
| *Yes* | 491 (27.3) | 113 (27.2) |
| Diet of mother |  |  |
| *Conventional* | 1885 (67.1) | 294 (62.8) |
| *Organic (incl. biodynamic)* | 923 (32.9) | 174 (37.2) |
| Diet of child: |  |  |
| *Conventional (≤25% organic)* | 1554 (81.4) | 360 (80.2) |
| *Organic (incl. biodynamic) (>25% organic)* | 355 (18.6) | 89 (19.8) |
| Vitamin D/AD supplementation during the first 2 years of life |  |  |
| *No* | 466 (18.8) | 96 (20.6) |
| *Yes* | 2011 (81.2) | 370 (79.4) |
| Vegetarian diet of child |  |  |
| *No* | 1943 (96.0) | 419 (94.4) |
| *Yes* | 82 (4.0) | 25 (5.6) |
| Regular intake of milk |  |  |
| *No* | 490 (25.3) | 107 (26.5) |
| *Yes* | 1445 (75.3) | 347 (73.5) |
| Regular intake of organic milk |  |  |
| *No* | 641 (75.3) | 152 (87.5) |
| *Yes* | 210 (24.7) | 59 (12.5) |
| Regular intake of yogurt |  |  |
| *No* | 464 (24.4) | 110 (28.4) |
| *Yes* | 1438 (75.6) | 338 (71.6) |
| Regular intake of organic yogurt |  |  |
| *No* | 619 (71.5) | 157 (87.5) |
| *Yes* | 247 (28.5) | 59 (12.5) |
| Regular intake of cheese |  |  |
| *No* | 510 (27.1) | 114 (30.1) |
| *Yes* | 1370 (72.9) | 330 (69.9) |
| Regular intake of organic cheese |  |  |
| *No* | 740 (87.2) | 186 (94.1) |
| *Yes* | 109 (12.8) | 28 (5.9) |
| Regular intake of organic fruit |  |  |
| *No* | 1738 (85.5) | 376 (84.5) |
| *Yes* | 295 (14.5) | 69 (15.5) |
| Regular intake of organic raw vegetables: |  |  |
| *No* | 650 (74.8) | 155 (71.4) |
| *Yes* | 219 (25.2) | 62 (28.6) |

^a^ Totals may not add up to the total group size due to missing values; ^b^ Data unavailable for the complete cohort (based on home visit/fecal sampling); ^c^ Lower: primary school, preparatory vocational or lower general secondary school. Middle: vocational, higher general secondary or pre-university. High: higher vocational or academic education ^d^ Never: Child did not use a pacifier. Early: Use of pacifier recorded at 3 and/or 7 months of age. Late: First use of pacifier recorded at 24 months or long term use of pacifier from 3 and/or 7 months until 24 months.

**Supplementary Table 3.** Multivariable logistic regression model showing associations between potential determinants and *Methanobrevibacter smithii* presence.

| **M. smithii presence** | | | |
| --- | --- | --- | --- |
| **Main effects model (N=419)** | | | |
| **Determinant** | **OR (CI95%)** | **FDR crit.^a^** | **p-value** |
| Regular intake of organic milk^b, c^ |  |  |  |
| *No* | Ref. |  |  |
| *Yes* | 5.73 (1.92; 17.03) | 0.010 | **0.002^d^** |
| Regular intake of organic yogurt^b, c^ |  |  |  |
| *No* | Ref. |  |  |
| *Yes* | 4.40 (1.60; 12.08) | 0.010 | **0.004^d^** |
| Diet of child^e^ |  |  |  |
| *Conventional*  *(≤25% organic)* | Ref. |  |  |
| *Organic (incl. biodynamic) (>25% organic)* | 0.37 (0.18; 0.78) | 0.020 | **0.009^d^** |
| *Fiber intake (g)*^e^ | 0.96 (0.90; 1.03) | 0.040 | 0.256 |
| *Age at fecal sampling (years)*^e^ | 0.83 (0.64; 1.09) | 0.030 | 0.186 |

*^a^* Critical FDR cut-off level as determined by Benjamini-Hochberg procedure; *^b^* Missing value category omitted (included in FDR correction); *^c^* Due to multicollinearity both regular intake of organic milk and regular intake of organic yogurt are reported. Both were run in a separate model; *^d^* significant association after correction for FDR by Benjamini-Hochberg procedure; *^e^* Parameter estimates presented for the model containing organic milk intake. Parameter estimates did not change substantially when the variable ‘organic milk intake’ was replaced by the variable ‘organic yoghurt intake’ (data not shown).

**Supplementary Table 4.** Secondary multivariable logistic regression model showing associations between potential determinants and the presence of *Methanobrevibacter smithii*.

| **M. smithii presence** | | | |
| --- | --- | --- | --- |
| **Main effects model (N=406)** | | | |
| **Determinant** | **OR (CI95%)** | **FDR crit.^a^** | **p-value** |
| First exposure to antibiotics (during first 2 years of life) |  |  |  |
| *Never* | Ref. |  |  |
| *At 0-7 months* | 0.57 (0.31; 1.04) | 0.030 | 0.065 |
| *At 8-12 months* | 1.20 (0.58; 2.48) | 0.050 | 0.623 |
| *At 13-24 months* | 0.80 (0.40; 1.59) | 0.040 | 0.519 |
| Regular intake of organic products (cheese, milk and yogurt) |  |  |  |
| *Trend (0, 1, 2, 3)* | 2.10 (1.32; 3.33) | 0.010 | **0.002^b^** |
| Diet of child |  |  |  |
| *Conventional*  *(≤25% organic)* | Ref. |  |  |
| *Organic (incl. biodynamic) (>25% organic)* | 0.32 (0.16; 0.67) | 0.020 | **0.003^b^** |

*^a^* Critical FDR cut-off level as determined by Benjamini-Hochberg procedure; *^b^* Significant association after correction for FDR by Benjamini-Hochberg procedure.

**Supplementary Table 5.** Multivariable linear regression model showing associations between potential determinants and the presence of *Methanobrevibacter smithii* abundance.

| **M. smithii abundance (log10 DNA copies per gram feces)** | | | |
| --- | --- | --- | --- |
| **Main effects model (N=431)** | | | |
| **Determinant** | **β (CI95%)** | **FDR crit.^a^** | **p-value** |
| Younger and older siblings (at 4-5 years of age) |  |  |  |
| *None* | Ref. |  |  |
| *One* | -0.88 (-1.91; 0.15) | 0.029 | 0.094 |
| *Two or more* | -0.99 (-2.07; 0.09) | 0.021 | 0.072 |
| First exposure to antibiotics (during first 2 years of life) |  |  |  |
| *Never* | Ref. |  |  |
| *At 0-7 months* | -0.81 (-1.65; 0.02) | 0.017 | 0.056 |
| *At 8-12 months* | 0.16 (-0.71; 1.03) | 0.042 | 0.717 |
| *At 13-24 months* | 0.27 (-0.61; 1.14) | 0.038 | 0.551 |
| Monthly exposure to farm (animals) at 6-7 years of age^b^ |  |  |  |
| *No* | Ref. |  |  |
| *Yes* | -0.50 (-1.26; 0.25) | 0.033 | 0.192 |
| Presence of pets at 6 to 7 years of life |  |  |  |
| *None* | Ref. |  |  |
| *Dog* | 0.12 (-0.99; 1.23) | 0.050 | 0.829 |
| *Cat* | 0.14 (-0.84; 1.12) | 0.046 | 0.774 |
| *Other* | 1.03 (0.20; 1.86) | 0.004 | 0.016 |
| *Combination* | 0.91 (-0.10; 1.91) | 0.025 | 0.076 |
| *Breastfeeding (months)* | 0.07 (0.00; 0.15) | 0.013 | 0.051 |

*^a^* Critical FDR cut-off level as determined by Benjamini-Hochberg procedure; *^b^* Missing value category omitted (included in FDR correction)

**Supplementary Table 6.** Final multivariable linear regression model showing associations between potential determinants and abundance of *Methanobrevibacter smithii*.

| **M. smithii abundance (log10 DNA copies per gram feces)** | | | |
| --- | --- | --- | --- |
| **Adjusted main effects model (N=431)^a^** | | | |
| **Determinant** | **β (CI95%)** | **FDR crit.^b^** | **p-value** |
| Younger and older siblings  (at 4-5 years of age) |  |  |  |
| *None* | Ref. |  |  |
| *One* | -0.89 (-1.94; 0.16) | 0.021 | 0.095 |
| *Two or more* | -1.04 (-2.14; 0.06) | 0.011 | 0.065 |
| First exposure to antibiotics (during first 2 years of life) |  |  |  |
| *Never* | Ref. |  |  |
| *At 0-7 months* | -0.81 (-1.65; 0.04) | 0.009 | 0.062 |
| *At 8-12 months* | 0.17 (-0.70; 1.05) | 0.035 | 0.697 |
| *At 13-24 months* | 0.26 (-0.63; 1.14) | 0.029 | 0.568 |
| Monthly exposure to farm (animals) at 6-7 years of age^c^ |  |  |  |
| *No* | Ref. |  |  |
| *Yes* | -0.50 (-1.26; 0.27) | 0.024 | 0.200 |
| Presence of pets at 6 to 7 years of life |  |  |  |
| *None* | Ref. |  |  |
| *Dog* | 0.13 (-0.99; 1.25) | 0.044 | 0.821 |
| *Cat* | 0.15 (-0.84; 1.14) | 0.041 | 0.767 |
| *Other* | 1.04 (0.20; 1.88) | 0.003 | 0.015 |
| *Combination* | 0.94 (-0.08; 1.96) | 0.018 | 0.070 |
| *Breastfeeding (months)* | 0.08 (-0.01; 0.15) | 0.015 | 0.065 |

*^a^* Model adjusted for: age at fecal sampling (years), gender (male/female), recruitment group (conventional/alternative), total energy intake (kcal) and BMI (z-score); *^b^* Critical FDR cut-off level as determined by Benjamini-Hochberg procedure; *^c^* Missing value category omitted (included in FDR correction)

**Supplementary Table 7.** Main logistic regression model showing associations between potential determinants and presence of *Methanosphaera stadtmanae*.

| **M. stadtmanae presence** | | | |
| --- | --- | --- | --- |
| **Main effects model (N=420)** | | | |
| **Determinant** | **OR (CI95%)** | **FDR crit.^a^** | **p-value** |
| *Birthweight (g)*^b^ | 1.001 (1.000; 1.002) | 0.027 | 0.061 |
| Place and mode of delivery^b^ |  |  |  |
| *Natural birth at home* | Ref. |  |  |
| *Natural/artificial birth at hospital* | 1.49 (0.64; 3.46) | 0.041 | 0.350 |
| *Caesarean section* | 5.14 (1.64; 16.07) | 0.005 | 0.005 |
| First exposure to antibiotics (during first 2 years of life)^b^ |  |  |  |
| *Never* | Ref. |  |  |
| *At 0-7 months* | 0.60 (0.19; 1.95) | 0.046 | 0.395 |
| *At 8-12 months* | 0.70 (0.22; 2.28) | 0.050 | 0.552 |
| *At 13-24 months* | 2.72 (1.13; 6.57) | 0.023 | 0.026 |
| Regular intake of organic milk^c, d^ |  |  |  |
| *No* | Ref. |  |  |
| *Yes* | 2.06 (0.66; 0.96) | 0.029 | 0.214 |
| Regular intake of organic yogurt^c, d^ |  |  |  |
| *No* | Ref. |  |  |
| *Yes* | 1.69 (0.49; 5.86) | - | 0.680 |
| *Breastfeeding (months)*^b^ | 1.06 (0.96; 1.16) | 0.036 | 0.237 |
| Regular intake of organic fruit^b^ |  |  |  |
| *No* | Ref. |  |  |
| *Yes* | 3.06 (1.17; 8.03) | 0.018 | 0.023 |
| *Animal protein intake (en%)*^b^ | 0.81 (0.69; 0.96) | 0.009 | 0.014 |

*^a^* Critical FDR cut-off level as determined by Benjamini-Hochberg procedure; *^b^* Parameter estimates presented for the model containing organic milk intake. Parameter estimates did not change substantially when the variable ‘organic milk intake’ was replaced by the variable ‘organic yoghurt intake’ (data not shown); *^c^* Missing value category omitted (included in FDR correction); *^d^* Due to multicollinearity both regular intake of organic milk and regular intake of organic yogurt were included in separate models, which included all other variables as listed in this table.
